# Assessing Health Behaviors as Predictors of Psychological Resilience and BMI in a National and Small Military College Sample

**DOI:** 10.3390/healthcare13222877

**Published:** 2025-11-12

**Authors:** Kylie Blodgett, Rachele Pojednic

**Affiliations:** 1 Department of Health and Human Performance, Norwich University, Northfield, VT 05663, USA; kcowens@norwich.edu; 2 Nutrition and Health Innovation Research Institute, School of Medical and Health Sciences, Edith Cowan University, Joondalup, WA 6027, Australia; 3 Stanford Lifestyle Medicine, Stanford Prevention Research Center, Stanford University School of Medicine, Palo Alto, CA 94305, USA

**Keywords:** diet, exercise, physical activity, military, sleep, college, resilience

## Abstract

**Highlights:**

**What are the main findings?**

- First-year students at a military college engage in more vigorous physical activity and strength training compared to a representative national sample.
- In the national sample, higher levels of physical activity, strength training, and better sleep are associated with greater psychological resilience and healthier BMI values and these associations were not observed in the military college students.

**What are the implication of the main findings?**

- Promoting physical activity and adequate sleep may enhance resilience and support healthy body weight in civilian college populations.
- Military training may overshadow the influence of individual health behaviors on resilience and BMI, suggesting that structured programs may interact differently with health outcomes compared to civilian settings.

**Abstract:**

**Objective:** Assess potential associations between the health behaviors, resilience, and body mass index (BMI) of first-year students and compare these metrics between students at a small military college and a representative national sample. **Methods:** Cross-sectional data were collected via an online survey administered by the American College Health Association (ACHA) during the fall of 2022 from first-year students at a senior military college (n = 77) and first-year college students who completed the NCHA-III (n = 7644). **Results:** Military college students had significantly higher levels of weekly vigorous physical activity and strength training than the national sample. Vigorous physical activity, strength training, and better sleep significantly predicted improved psychological resilience and BMI values in the national sample. No behaviors predicted psychological resilience or BMI in the military college sample. **Conclusions:** Health behaviors like physical activity and sleep may improve resilience and body weight in civilian college students. However, combining military training with college life appears to have less impact on the relationship between health behaviors and resilience.

## 1. Introduction

Obesity is one of the leading factors limiting military service eligibility in the United States, making elevated body mass index (BMI) not only a public health and economic challenge, but also a pressing concern for national security [1,2,3]. In 2019, obesity-related medical care costs exceeded USD 173 billion, with the Department of Defense spending approximately USD 1.5 billion annually to address obesity among active-duty service members [4]. As military service typically begins in late adolescence and early adulthood, understanding the behavioral and environmental factors that drive obesity and obesity-related lifestyle behaviors during this time period is essential to mitigate related care expenditures while preserving national security interests. Emerging adulthood [aged 18–24], a developmental stage characterized by identity exploration and instability [5], is a period marked by significant psychosocial, academic, and occupational transitions that often coincide with elevated stress, an established moderate-to-severe contributor to obesity development [6,7]. Physiological markers of stress [8,9] and self-reported psychometric stress are related to obesity through poor dietary [10] and disordered eating behaviors [10,11], impaired sleep quality [12], and reduced physical activity [13]. This is likely true across the diverse learning and training environments that emerging adults may experience during this time period, including civilian college settings and athletic participation, senior military colleges (SMCs) and Reserve Officer Training Corps (ROTCs) programs, each of which differs in structure and obligations as students navigate increasing autonomy and responsibility [14,15]. Importantly, diet and physical activity behaviors that are established during this emerging adult period are known to persist into adulthood [16,17,18] and play a role in long-term care costs.

One moderator of stress is resilience [14,15,19], with psychological resilience specifically, defined as the ability to successfully manage and adapt to a variety of stressors [20]. Resilience may influence how emerging adults respond to stress and how likely they are to practice maladaptive health behaviors, particularly those related to obesity. In civilian college students, psychological resilience has been associated with both healthy and maladaptive eating behaviors [10,11], overall physical activity behaviors [21,22,23], and sleep quality [24]. Much of the research on resilience in military populations has focused on active-duty service members or previously deployed veterans, with outcomes typically in the context of physical resilience or job-related demands rather than psychological resilience. However relationships between resilience and behaviors such as sleep, diet, and physical activity have been established and are currently of high priority in military settings [25]. Prior to active-duty service, there is also a growing body of literature examining resilience in student veterans and military-affiliated college students, including those enrolled in SMCs or in ROTC programs. These studies highlight that resilience in student veterans is shaped not only by exposure to stress, but also by military identity, prior service experience, and the process of socialization into military-affiliated educational programs [14,15,26]. Importantly, student veterans and SMC/ROTC cadets may experience unique stressors and supports compared to civilian students, such as balancing academic demands with military obligations, which may influence resilience pathways and related health behaviors [27]. Gender differences can also intersect with these military-affiliated experiences, as resilience, BMI, and health behaviors can vary by sex, and prior research on women veterans suggest distinct mechanisms that may shape health outcomes in higher education settings [15].

Because resilience may act as a protective factor against stress-related behaviors associated with obesity, understanding its role in military-affiliated college students is an important step toward developing novel, more effective interventions that could potentially impact long-term costs and issues of national security readiness. Current approaches to reduce and prevent the development of obesity in college and military training settings generally focus on strategies to improve knowledge and facilitate the environmental changes necessary for improving diet and exercise behaviors at the individual and interpersonal levels. Examples of these interventions include education in the classroom [25,28,29,30], facilitating social support in behavior change [25,29,30], or altering the food environment [28,31]. Results from these interventions are underwhelming and suggest a persisting need to identify novel approaches for improving health behaviors in young adult and military populations to prevent the development of obesity and improve acute health and preparedness for service. Furthermore, it is critical to understand the unique needs of college and military populations as they develop persistent health behaviors, and how those needs may guide targeted prevention for both civilian and military settings. The purpose of this descriptive study was to (1) assess how well the health behaviors, resilience, and BMI of first-year students at a small, rural senior military college with cadet and civilian lifestyles compare to a representative national sample of civilian college students, and (2) assess potential associations between health behaviors, resilience, and BMI in the national and small military college samples. We hypothesized that military-affiliated students would differ in resilience and obesity-related lifestyle behaviors compared to their civilian peers and that resilience would be associated with BMI and modifying behaviors.

## 2. Materials and Methods

### 2.1. Participants

First-year students at a small senior military college (SMC) were recruited via email and provided electronic informed consent to take the National College Health Assessment version 3 [NCHA-III]. Participants were included if they were at least 18 years old on the day of the survey’s launch. Participants were excluded if they were [a] dual-enrolled students completing their last year of high school at the college, [b] study abroad students from a different country enrolling for a short time, or [c] study away students from a different US college enrolling for a short time. The participants represented three lifestyles on campus: (1) civilian students (CIVs) who live a traditional college lifestyle with no military training or obligations; (2) corps of cadets students (CORPSs) who participate in a military lifestyle, including weekly physical training, living in military-style barracks, wearing a uniform, and living within a student leadership chain of command; and (3) Reserve Officer Training Corps students (ROTCs) who lead a similar lifestyle to CORPS and have or are pursuing a scholarship and contract with one of the military service branches. In addition to the responsibilities of the CORPS, they also have additional military and physical training requirements with the ROTC units on campus. To assess the generalizability of this cohort to other college students, data from the national sample of the NCHA survey collected during the fall of 2022 was obtained from the American College Health Association (ACHA), and data from self-reported first-year college students were included to remain consistent with the inclusion of only first-year students in the SMC sample. The SMC cohort was compared to the national cohort of freshmen because the SMC institution does not represent the academic or demographic characteristics of regional or small college populations.

### 2.2. Design

The NCHA-III was administered during the first three weeks of the 2022 fall semester to incoming first-year college students at a small, rural senior military college in New England. Students who satisfied the inclusion criteria were sent an email from the ACHA with a link to the NCHA-III survey. Participants used the ACHA link to consent and take the survey, which is described below. To assess generalizability, measures of BMI, diet, physical activity, sleep, and resilience were compared between the NCHA and SMC samples. To assess differences by lifestyle, these metrics were also compared across the CIV, CORPS, and ROTC cohorts. The study was approved by the Norwich University Institutional Review Board (Approved Protocol #8).

### 2.3. Measures

The NCHA-III [32] is a comprehensive survey administered by the ACHA and focused on measuring the health habits, behaviors, and perceptions of college students. It is administered at an average of n = 76.8 colleges and universities nationally each spring and fall semester to an average of n = 50,197 students per semester [33] and embeds seven validated instruments. To assess the impacts of health behaviors on BMI in first-year college students in the studied cohort, we only analyzed individual questions that addressed BMI, diet quality, sleep quality, and physical activity. Height and weight were self-reported, and BMI was calculated as kg/m^2^. Regarding specific survey questions, we included three questions about diet quality, which assessed daily intake of sugar-sweetened beverages (SSBs), fruits, and vegetables. The daily intake of SSBs was measured continuously as the number of self-reported beverages consumed per day over the past 7 days, while the daily intake of fruits and vegetables were measured as categorical variables, with the options of 0 servings/day, 1–2 servings/day, 3–4 servings/day, 5–6 servings/day, or more than 6 servings/day. The total amount of moderate and vigorous physical activity performed in the past 7 days were measured continuously as the number of minutes per day over the past 7 days, while the number of days participating in strength training was measured as a categorical variable with the options of 0 days, 1 day, 2 days, 3 days, 4 days, 5 days, 6 days, and 7 days. Sleep quality was assessed categorically across five questions, as follows: “on how many of the last 7 days did you: (1) Wake up too early in the morning and couldn’t get back to sleep? (2) Feel tired or sleepy during the day? (3) Have an extremely hard time falling asleep? (4) Get enough sleep so that you felt rested? (5) Take a nap?” Response options included 0 days, 1 day, 2 days, 3 days, 4 days, 5 days, 6 days, and 7 days. We used the responses to these questions to make a sleep quality score by reverse coding question 4, “on how many of the last 7 days did you get enough sleep so that you felt rested?” and summing the responses to all 5 questions. This created an overall continuous score representing sleep quality, with a higher score representing poorer sleep quality. To assess the impacts of these health behaviors on resilience, we used the questions summarized above regarding diet, physical activity, and sleep, and added the scored two-item version of the Connor–Davison resilience scale, which specifically assesses adaptability [34]. Finally, we added the PrimeScreen, a validated food frequency tool, to the SMC administration of the NCHA-III survey, allowing for a more comprehensive assessment of overall diet quality [35]. The PrimeScreen was not administered as part of the national NCHA-III administration.

### 2.4. Data Analysis

Data were analyzed using the IBM SPSS Statistics for Mac, Version 29.0. Demographic characteristics were summarized using descriptive statistics. Data were assessed for normality in the NCHA and SMC samples. Correlations between diet variables, physical activity variables, sleep, resilience, and BMI were performed at the NCHA and SMC sample levels using Spearman’s rank coefficient. Due to a lack of normality in several of the continuous variables and the inclusion of several ordinal variables, Mann–Whitney U tests were applied to assess potential differences in all variables between the NCHA first-year college students and the SMC sample. Differences were also assessed among the various SMC military college lifestyles, including ROTC, CORPS, and CIV, using the Kruskal–Wallis test with Bonferroni corrections. Multiple linear regression was used to assess diet behaviors, physical activity behaviors, and sleep quality as potential predictors of two dependent variables, BMI and resilience. Additional confounders were added in the linear regression models run on the SMC cohort to understand if the findings in this small sample were due to behavioral variables or outside factors such as race, biological sex, socioeconomic status, or GPA. Diet comparisons between the SMC and NCHA samples were conducted using fruit, vegetable, and SSB intake only. Diet comparisons between the ROTC, CORPS, and CIV lifestyles within the SMC sample were performed using fruit, vegetable, and SSB intake, as well as overall diet quality assessed via the PrimeScreen.

## 3. Results

### 3.1. Sample Characteristics

Of the NCHA respondents (n = 33,774), only self-reported first-year college students [n = 7644] were included for analysis. The sample was predominately female and Caucasian [Table 1]. SMC participants were recruited via email (n = 624), and respondents (n = 77, 12.34% response rate) were predominately male and Caucasian (Table 1), with CIV (n = 34), CORPS (n = 18), and ROTC (n = 25) represented. This sample is demographically representative of the institution, except for Asian/Asian American students, who represent a larger proportion of the sample than the student population [36]. The SMC sample contained a higher proportion of males, Caucasians, and Asian and Asian Americans than the NCHA sample.

### 3.2. Associations Between Behavioral Variables, BMI, and Resilience

Table 2 summarizes Spearman’s rho correlation values for behavioral variables, BMI, and resilience in the NCHA and SMC participants. Moderate and vigorous physical activity, strength training, fruit intake, and vegetable intake were all positively correlated with resilience (*p* < 0.001), while sugar-sweetened beverage intake and poor sleep quality were negatively correlated with resilience (*p* < 0.001; Table 2). Moderate and vigorous physical activity exhibited negative associations with BMI, while sugar-sweetened beverage intake and poor sleep quality exhibited positive associations with BMI.

Table 3 summarizes Spearman’s rho correlation values for the behavioral variables, BMI, and resilience in SMC participants. None of the behavioral variables were significantly associated with BMI in this population, and only vigorous physical activity was significantly associated with resilience, exhibiting a positive correlation (*p* < 0.05). Resilience and BMI were not significantly associated with one another in either the NCHA or the SMC samples.

### 3.3. Behavioral and Outcome Variable Comparisons Between the NCHA and SMC Participants

Table 4 presents the mean ± standard deviation for all behavioral and outcome variables between the NCHA and SMC first-year college students. The Mann–Whitney U test results indicated that weekly vigorous activity (*z* = −5.499, *p* < 0.001), days of strength training (*z* = −7.378, *p* < 0.001), and daily servings of fruit (*z* = −2.110, *p* = 0.035) were significantly higher in the SMC than in the NCHA participants. There were no significant differences in weekly moderate PA (*z* = −1.375, *p* = 0.169), daily servings of sugar-sweetened beverages (*z* = −0.541, *p* = 0.589), daily servings of vegetables (*z* = −1.921, *p* = 0.055), poor sleep quality (*z* = −1.852, *p* = 0.064), resilience (*z* = −1.449, *p* = 0.147), or BMI (*z* = −1.406, *p* = 0.160) between the NCHA and SMC participants.

### 3.4. Predictors of BMI and Resilience in the NCHA Participants

Multiple linear regression was used to assess the combined effects of exercise, diet, and sleep behaviors on BMI and resilience in the NCHA sample. The first model assessed the influence of the independent variables of moderate PA, vigorous PA, strength training, SSB intake, fruit intake, vegetable intake, and poor sleep quality on BMI. The model was significant, F[7, 7204] = 18.1, *p* < 0.001, and explained 1.6% of the variance (r^2^ = 0.016) in BMI. Vigorous PA (β = −0.001, *p* < 0.001) and strength training (β = −0.082, *p* = 0.021) were significant negative predictors of BMI, while SSB intake (β = 0.064, *p* < 0.001) and poor sleep quality (β = 0.074, *p* < 0.001) were significant positive predictors of BMI.

The second model assessed the influence of the same independent variables on resilience. The model was significant (F[7, 7394] = 85.151, *p* < 0.001) and explained 7.4% of the variance in resilience (r^2^ = 0.074). Vigorous PA (β = 0.000), *p* < 0.001), strength training (β = 0.090, *p* < 0.001), and servings of vegetables (β = 0.122, *p* < 0.001) were significant positive predictors of resilience, and poor sleep quality (β = −0.050, *p* < 0.001) was a significant negative predictor of resilience.

### 3.5. Behavioral and Outcome Variable Comparisons Among the CIV, CORPS, and ROTC Groups

Table 5 presents the mean± standard deviation for all behavioral and outcome variables in the ROTC, CORPS, and CIV participants in the SMC sample. A Kruskal–Wallis test indicated significant differences in resilience based on lifestyle (H[2] = 14.044, *p* < 0.001). Pairwise comparisons suggested that the ROTC participants had higher resilience than the CORPS [*p* = 0.001] and CIV participants (*p* = 0.020). This was the only difference noted between the ROTC and CORPS participants. Weekly vigorous activity was different among groups (H[2] = 22.266, *p* < 0.001), with greater minutes reported in the ROTC (*p* = 0.000) and CORPS groups (*p* = 0.027) than in the CIV group. The number of days of strength training was also different among groups (H[2] = 26.528, *p* < 0.001), with more days reported in the ROTC (*p* < 0.001]) and CORPS groups (*p* < 0.001) than in the CIV group.

There were no significant differences in the minutes of moderate physical activity (H[3] = 5.487, *p* = 0.064), daily servings of sugar-sweetened beverages (H[3] = 2.484, *p* = 0.289), daily servings of fruit (H[3] = 5.689, *p* = 0.058), daily servings of vegetables (H[3] = 0.906, *p* = 0.636), diet quality (H[3] = 3.104, *p* = 0.212), poor sleep quality (H[3] = 0.122, *p* = 0.941), or BMI (H[3] = 0.973, *p* = 0.615) between the ROTC and CIV participants.

Because there were no behavioral differences in the CORPS and ROTC participants, we collapsed participants into one group representing military training participants and compared all health behaviors between this group and the CIV group. Weekly moderate PA (z = −2.222, *p* = 0.026), vigorous PA (z = −4.525, *p* < 0.001), and days of strength training (z = −5.038, *p* < 0.001) were all significantly higher in the military training group than in the CIV group. There were no significant differences in any of the other health behaviors between these two groups.

### 3.6. Predictors of BMI and Resilience in the SMC Participants

Multiple linear regression was used to assess the combined effects of behavioral variables and potential confounders on BMI and resilience in the SMC sample. The first model assessed the influence of the confounders of race, gender, parent education as a proxy for socioeconomic status, and GPA, and the independent variables of moderate PA, vigorous PA, strength training, SSB intake, fruit intake, vegetable intake, overall diet quality, and poor sleep quality on BMI. The model was not significant (F[12, 52] = 1.478, *p* = 0.163), although race (β = 0.978, *p* = 0.014) and parent education (β = −0.869, *p* = 043) were significant independent predictors of BMI. No behavioral variables were significant. The second model assessed the influence of the same confounding and independent variables on resilience, and was not significant (F[12, 54] = 1.278, *p* = 0.258). None of the independent or confounding variables were significant predictors of resilience. To assess the impact of the behavioral variables alone, the confounders were removed and the model re-run. The model remained insignificant (F[8, 58] = 1.184, *p* = 0.325).

The diet quality data in the SMC cohort allowed us to run additional analyses in this cohort that we could not run in the NCHA cohort. Based on the Spearman’s correlation results, it appears that diet quality is associated with SSB intake, moderate PA, and days of strength training. Therefore, a third model was run to assess those three independent variables as potential predictors of overall diet quality. The model was significant (F[3, 65] = 3.395, *p* = 0.023) and explained 13.5% of the variance in overall diet quality. Days of strength training was the only significant predictor (β = 0.598, *p* = 0.037), and increases in strength training predicted poorer diet quality.

## 4. Discussion

One of the major aims of this study was to assess how well the health behaviors, BMI, and resilience in first-year SMC students compared to those of first-year NCHA students. Our analyses suggest that first-year SMC students differ from first-year NCHA students in key health behaviors. Notably, first-year SMC students had more minutes of vigorous physical activity and days of strength training per week, and a higher daily fruit intake than NCHA students. Health behaviors including vigorous physical activity, strength training, and sleep significantly predicted BMI and resilience in the NCHA sample, and resilience was an independent predictor of BMI. None of the health behaviors, alone or in combination, predicted BMI or resilience in the SMC sample. When looking at these behaviors by the various lifestyles in the small college setting, we found that CIVs had lower levels of vigorous physical activity and strength training than their CORPS and ROTC peers, and lower resilience than their ROTC peers. These findings suggest two key things. First, the difference in lifestyle between civilian college students and those training for future military obligations may uniquely influence the development of health behaviors. Second, psychological resilience may serve as a positive mediator between healthy behaviors and obesity in college students who are not training for the military, while military students may be developing resilience via different mechanisms that do not necessarily interact with health behaviors.

### 4.1. Differences Between Civilian Students and Military Trainees at an SMC

One key difference between students who live a military lifestyle and students who live a more traditional college lifestyle is the amount of physical activity they perform. We found that the amounts of weekly vigorous physical activity and strength training were significantly higher in both ROTC and CORPS participants than in CIV participants. This difference makes sense because of the physical training requirements that come with both CORPS and ROTC participation in the military college setting. The CIV students are not required to participate in any physical activity, like most traditional college students, and may experience a variety of barriers to physical activity, including time, comfort, self-efficacy, and social factors [6]. Although unsurprising, this finding provides important context for why the SMC sample had significantly higher physical activity levels than the NCHA sample. This may further explain why vigorous physical activity and strength training did not predict BMI or resilience in the SMC sample, as it did in the NCHA sample. Had we recruited a larger subset of CIV participants, we may have seen that the CIV students followed similar behavioral patterns as the NCHA sample, supporting the idea that participating in military training has a differential impact on the development of health behaviors and obesity risk in college students. Alternatively, the parameters around physical activity and BMI in the military setting may have attenuated the relationships between vigorous physical activity, strength training, resilience, and BMI. If this explanation were plausible, we would likely see less variance in the physical activity and BMI metrics; however, the variance in the physical activity variables is greater in the SMC sample compared to the NCHA sample, and the variance in BMI is similar.

Data from the SMC cohort also indicate differences in diet quality by student lifestyle. Fruit intake was lower in the CORPS than in the CIV participants, and PrimeScreen results showed that overall diet quality was significantly lower in the ROTC than in the CIV students. When assessing moderate activity, vigorous activity, and days of strength training as predictors of diet quality, we found that vigorous activity and strength training were significant negative predictors, suggesting that higher activity levels may be associated with poorer diet quality in the CORPS and ROTC students. While food quality on military bases has been a concern [37,38], all students in this study had access to the same cafeteria and food options, suggesting that these differences are likely not due to differences in food availability. There is evidence that diet quality is poor in military personnel during basic training, including inadequate intake of macro- and micronutrients compared to the Military Dietary Reference Intakes [MDRI] [39,40]. In one small cohort of male ROTC cadets, only 15% of the participants met the MDRI for calories, with 62% demonstrating clinically low energy availability [41]. These findings illustrate that high physical training demands may negatively impact diet quality and align with the current study outcomes, potentially due to a shift in dietary priorities towards ready-to-eat energy-dense foods and away from nutrient-dense options. Unfortunately, the questions used to assess diet quality in the NCHA-III survey are limited to fruit, vegetable, and sugar-sweetened beverage intake, limiting the comparison of diet quality from the PrimeScreen with the current sample. A consistent, comprehensive assessment of diet quality in a larger sample size of military and non-military students is needed. Nevertheless, the findings from the SMC cohort suggest that while a military college lifestyle may increase physical activity levels, it may concurrently reduce diet quality compared to the civilian student cohort. 

Finally, we identified a difference in psychological resilience by SMC student lifestyle, with significantly higher resilience in the ROTC participants than in the CORPS or CIV participants. Although it was surprising to find a difference in resilience between the ROTC and CORPS groups, who both live similar military lifestyles while on campus, it is important to consider that the difference between the students who join the ROTC and the corps of cadets is unique. ROTC participants are actively pursuing a contract in the military as future officers and likely have a scholarship with a branch of the United States military, which upholds stringent selection criteria that assess academic success, physical abilities, and leadership skills, all of which may require greater resilience [42]. Although CORPS students are living a similar military lifestyle while on campus, they are not currently pursuing an ROTC scholarship or military contract and have not been screened for skills and successes that may predict higher levels of resilience. The differences between the ROTC and CORPS students mirror findings that resilience is typically higher in active-duty officers than in enlisted personnel, a distinction demonstrated to be largely driven by differences in screening and training [37,43]. Similarly, SMC ROTC students, who resemble future officers in education and leadership preparation, exhibit greater resilience, suggesting that preparedness and training may play a role.

### 4.2. The Role of Resilience in the Relationship Between Health Behaviors and Obesity

Although health behaviors did not predict BMI or resilience in the SMC sample, we did find that vigorous physical activity, strength training, and sleep positively predicted resilience and negatively predicted BMI in the NCHA sample. Resilience was also an independent negative predictor of BMI in the NCHA sample, suggesting that health behaviors may improve resilience, which could play a role in the development of obesity in college students. Vigorous physical activity [44] and high amounts of overall physical activity [45] have been found to positively influence resilience in college students. Sleep has also been associated with resilience in this demographic [46]. There is further evidence that sleep, physical activity, and resilience may play individual or mediatory roles in the development of a variety of other concerns such as social anxiety [47], depression [48], and social adaptation [49] in college students. Taken together, there is existing evidence to suggest that physical activity and sleep may interact with resilience to influence college students’ health and well-being, and the interactions of these constructs in the development of obesity is worth exploring. Our findings specifically support the potential model that health behaviors influence resilience, which may in turn have an impact on BMI.

There is also evidence that diet quality may impact resilience [21]; however, that was not represented in these data. This could be due to the limited assessment of diet quality in the NCHA-III survey. There are other factors that influence overall diet quality, such as whole grain, dairy, protein, and processed food intake, as well as environmental and social factors. As previously noted, diet quality and behaviors need to be more comprehensively assessed before they can be adequately considered as potential predictors of resilience.

Although we did not find that any health behaviors predicted resilience or BMI in the SMC sample, this may be due to the smaller sample size [n = 77], which limits the ability to detect weaker relationships and increases the risk of a Type II error. By contrast, in the much larger NCHA sample, health behaviors accounted for a small portion of the variance, likely due to the granularity of the questions being asked about physical activity, diet, and sleep. Despite the small SMC sample size, we did see that more days of strength training predicted poorer diet quality, which contradicts findings in military populations where higher diet quality is associated with greater resilience [50,51] and more resistance training [50]. This discrepancy may reflect psychosocial factors, as the transition to college is accompanied by a significant disruption to social and emotional networks, particularly at mealtimes. Current evidence on the eating behaviors of military populations suggest that maladaptive eating behaviors are not developed while in the military, but are maintained in the military environment [52]. Studying these dynamics more comprehensively and in larger, diverse student samples may help clarify how training environments influence the development of health behaviors and their impact on readiness and performance.

### 4.3. Limitations

There are a few important limitations worth addressing. Notably, the SMC sample size was small and underpowered, increasing the risk for Type II error in the SMC analyses. There was a significant discrepancy in the proportion of males and females between the NCHA and SMC samples. Although this is characteristic of the military college setting, it is an important distinction that limits our ability to draw conclusions about how well data from the SMC cohort generalizes to a college population. We also used a cross-sectional design, limiting our ability to draw conclusions about potential cause-and-effect relationships. For example, it is possible that an underlying trait of resilience affects health behaviors, whereas our hypothesis suggests that health behaviors impact the development of resilience in this cohort. Additionally, mediation testing may have been useful in clarifying the nature of these relationships; however, group sample sizes were too small for this analytic approach. The convenience sampling approach used in the SMC setting led to a sample that is not perfectly aligned with the student body. The use of the PrimeScreen in the SMC sample allowed for conclusions about diet quality that could not be directly compared to the NCHA sample because of the lack of diet quality data in the larger survey administration. Finally, perceived and physiological stress are likely to play an important role in the relationships between resilience and health behaviors, and we did not have any stress data to assess this as a potential independent variable.

## 5. Conclusions

The current results demonstrate that in the national NCHA sample, physical activity and sleep predicted lower BMI and higher resilience, with resilience also independently predicting lower BMI. By contrast, these relationships were not observed in the smaller SMC cohort, though differences in physical activity, diet, and resilience across the CIV, ROTC, and CORPS students suggest that the traditional college and military college environments shape these behaviors and outcomes differently. The development of these behaviors and resilience over time warrants further attention in both college and military settings, as young adults develop persisting behavioral patterns in these settings that can affect their current and future health and fitness. Interventions to improve health behaviors and resilience, particularly in college military cadets, need to target these unique and nuanced differences to maximize military preparedness and fitness to serve. A military college that contains both traditional college and military lifestyles may provide a useful context in which to study the development of these relationships.

## Figures and Tables

**Table 1:** Demographic characteristics of the national first-year college students from the National College Health Assessment (NCHA) sample compared to senior military college (SMC) first-year students.

	NCHA [n = 7644]	SMC [n = 77]
Age	19.12 ± 4.10	18.51 ± 1.21
Biological Sex [female]	4994 [65.4%]	29 [37.3%]
Race White Hispanic/Latinx Asian/Asian American Black/African American Biracial/Multiracial American Indian/Native Alaskan Arab/Middle Eastern/North African Origin Native Hawaiian/Pacific Islander Other	5079 [66.4%] 1330 [17.4%] 984 [12.9%] 539 [7.1%] 364 [4.8%] 204 [2.7%] 117 [1.5%] 48 [0.6%] 73 [1.0%]	54 [70.1%] 5 [6.5%] 13 [16.9%] 5 [6.5%] 2 [2.6%] 2 [2.6%] 0 2 [2.6%] 3 [3.9%]

**Table 2:** Two-tailed Spearman’s rho values representing the correlation between behavioral variables, resilience, and BMI in the NCHA sample.

Outcome Measures	Moderate Physical Activity	Vigorous Physical Activity	Strength Training	SSB	Fruits	Vegetables	Sleep Quality
BMI	−0.041 **	−0.067 **	−0.018	0.064 **	0.007	0.016	0.066 **
Resilience	0.115 **	0.179 **	0.175 **	−0.052 **	0.069 **	0.099 **	−0.208 **

** indicates *p* < 0.001.

**Table 3:** Two-tailed Spearman’s rho values representing the correlation between behavioral variables, resilience, and BMI in the SMC sample.

Outcome Measures	Mod PA	Vig PA	Strength Training	SSB	Fruits	Vegetables	Diet Quality	Sleep Quality
BMI	0.093	0.077	0.123	−0.062	0.036	0.075	0.010	0.140
Resilience	147	0.299 *	0.183	−0.164	0.062	0.109	0.057	0.121

* indicates *p* < 0.05.

**Table 4:** Mean ± SD for all predictor and outcome variables in the NCHA participants compared to the SMC participants. Mann–Whitney U tests were used to compare the differences between groups.

	NCHA [n = 7644]	SMC [n = 77]
Moderate PA [min/wk]	265.81 ± 416.94	384.32 ± 578.96
Vigorous PA [min/wk]	138.82 ± 247.89	303.23 ± 451.796 **
Strength Training [days/wk]	1.92 ± 2.05	4.08 ± 2.52 **
SSB [servings/avg. day]	3.61 ± 5.19	4.41 ± 6.83
Fruit [servings/avg. day]	2.05 ± 0.68	2.22 ± 0.70 *
Vegetables [servings/avg. day]	2.15 ± 0.72	2.34 ± 0.74
Sleep ^1^	18.56 ± 6.20	19.48 ± 6.55
BMI [kg/m^2^]	24.20 ± 5.54	23.05 ± 5.72
Resilience ^2^	5.94 ± 1.60	5.64 ± 1.84

** *p* < 0.001, * *p* < 0.05, ^1^ continuous score based on the 5 sleep quality questions; higher score = poorer sleep, and ^2^ scale of 1–8; higher score = more resilience.

**Table 5:** Mean ± SD for all predictor and outcome variables in the ROTC, CORPS, and CIV participants from the SMC sample. Mann–Whitney U tests were used to compare the differences between groups.

	ROTC [n = 25]	CORPS [n = 18]	CIV [n = 34]
Moderate PA [min/wk]	500.92 ± 656.39	485.25 ± 762.90	251.09 ± 374.47
Vigorous PA [min/wk]	524.80 ± 641.09	329.38 ± 364.02	122.70 ± 151.58 **^,^***
Strength Training [days/wk]	5.68 ± 1.63	5.06 ± 1.55	2.38 ± 2.45 **^,^***
SSB [servings/avg. day]	3.88 ± 5.67	3.35 ± 7.03	5.33 ± 7.54
Fruit [servings/avg. day]	2.24 ± 0.66	1.89 ± 0.58	2.38 ± 0.74 ***
Vegetables [servings/avg. day]	2.36 ± 0.49	2.17 ± 0.62	2.41 ± 0.92
Diet Quality ^1^	41.95 ± 4.69	40.53 ± 4.39	38.81 ± 6.47
Sleep ^2^	17.32 ± 7.13	17.22 ± 6.09	17.71 ± 5.66
BMI [kg/m^2^]	23.97 ± 4.81	22.55 ± 3.88	22.58 ± 7.23
Resilience ^3^	6.67 ± 1.58	4.72 ± 1.74 *	5.39 ± 1.77 *^,^**

* ROTC vs. CORPS, *p* ≤ 0.05, ** ROTC vs. CIV, *p* ≤ 0.05, *** CORPS vs. CIV, *p* ≤ 0.05, ^1^ continuous score based on PrimeScreen; higher score = lower diet quality, ^2^ continuous score based on the 5 sleep quality questions, and ^3^ scale of 1–8; higher score = more resilience.

## Data Availability

De-identified data supporting the findings of this study are available from the corresponding author upon reasonable request due to the national dataset being shared with us under permission from ACHA.

## Abbreviations

The following abbreviations are used in this manuscript:

ACHA	American College Health Association
BMI	Body Mass Index
CIV	Civilian Student
CORPS	Corps of Cadets Student
NCHA	National College Health Assessment
ROTC	Reserve Officer Training Corps
SMC	Senior Military College
SSB	Sugar-Sweetened Beverages
